# Clinical diagnoses in young offspring from eastern Québec multigenerational families densely affected by schizophrenia or bipolar disorder

**DOI:** 10.1111/j.1600-0447.2007.01125.x

**Published:** 2008-02

**Authors:** M Maziade, N Gingras, N Rouleau, S Poulin, V Jomphe, M-E Paradis, C Mérette, M-A Roy

**Affiliations:** 1 Centre de recherche Université Laval Robert-Giffard QC, Canada; 2 École de psychologie, Université Laval, Québec QC, Canada

**Keywords:** high-risk offspring, schizophrenia, bipolar disorder, genetics, developmental precursor

## Abstract

Maziade M, Gingras N, Rouleau N, Poulin S, Jomphe V, Paradis M-E, Mérette C, Roy M-A. Clinical diagnoses in young offspring from eastern Québec multigenerational families densely affected by schizophrenia or bipolar disorder.

**Objective:**

The follow-up since 1989 of a large sample of multigenerational families of eastern Québec that are densely affected by schizophrenia (SZ) or bipolar disorder (BP) has permitted to look at the rates of DSM diagnoses in the young offspring of a SZ parent (HRSZ) and of a BP parent (HRBP) who had an extremely loaded family history.

**Method:**

The sample (average age of 17.5, SD 4.5) consisted of 54 high-risk offspring (HR) having one parent affected by a DSM-IV SZ or BP. The parents descended from 21 multigenerational families that constitute a quasi-total sample of such kindred in eastern Québec. The HRs were administered a lifetime best estimate DSM-IV diagnosis.

**Results:**

We observed that the rates, the diversity of diagnoses, the high comorbidity, the severity and the age of onset of the clinical diagnoses tended to be similar with those already reported in the offspring of affected parents with a low familial loading. Although the sample size was small, HRSZ and HRBP also tended to show similarities in their clinical status.

**Conclusion:**

Overall, taking into account methodological limitations, the observation early in life of some shared characteristics among HRSZ and HRBP in terms of non-psychotic diagnosis may be congruent with the accumulating evidence that several phenotypic features are shared in adulthood by the two major psychoses.

**Significant outcomes**

- Offspring at high genetic risk of major psychosis displayed early in life non-psychotic DSM disorders warranting a consultation.
- Various and highly comorbid disorders were observable in these offspring at extreme genetic risk.
- HRSZ and HRBP showed similarities in their clinical status.

**Limitations**

- Normal control group from the general population was absent.
- Small sample size may have prevented detection of differences between HRSZ and HRBP.
- Caution is required before generalizing findings to the offspring of an ill parent from the general population.

## Introduction

High-risk (HR) studies represent an informative method to investigate the genetic and environmental mechanisms leading to the development of schizophrenia (SZ) and bipolar disorder (BP). Given the neurodevelopmental aspects of these disorders, the investigation of the risk of behavioral deviancies during childhood, adolescence and postadolescence may help to understand the mechanisms that could explain the unresolved heterogeneity of SZ and BP. Moreover, HR studies may also throw light on the early mechanisms explaining the large number of epidemiological and genetic characteristics that are shared by SZ and BP (1–4). Only few cross-sectional or prospective HR offspring studies exist (5–10). We herein report on the clinical status of young offspring at extremely high risk of SZ and BP from the fourth and fifth generations descending from a large number of well-characterized and densely affected multigenerational families that represent a quasi-total sample of such pedigrees from the population of eastern Québec that we have followed up since 1989 (11–13). More specifically, in collaboration with all departments of psychiatry within our catchment area, we identified eligible kindreds through systematic screening of the medical archives and through contacts with experienced clinicians. After maintaining these systematic screening efforts for more than 10 years, we finally found ourselves in a position where we could not identify additional multi-affected kindreds.

### Recent studies of young offspring of SZ and BP reporting diagnoses in childhood and adolescence

High-risk offspring of parents with schizophrenia (HRSZ) or bipolar disorder (HRBP) are at higher risk of developing a major psychosis in adulthood but only recently have the studies investigated the type of non-psychotic diagnoses from which these children and adolescents may suffer early in their life. Most of the HR studies used behavioral scales instead of diagnostic instruments and targeted an affected parent whose family history was not documented. Studies of young offspring having used diagnostic categories now suggest that 40–60% of the HRs, be they HRSZ or HRBP, had, early in life, one or more non-psychotic diagnoses warranting a psychiatric consultation, that these diagnoses were often comorbid and encompassed diverse diagnostic categories (14–23).

For instance, in 41 HRSZ with a mean age of 17 years, Hans et al. (14) found a rate of more than 50% of lifetime DSM-IV diagnoses, a large proportion being comorbid. Among other diagnoses, 17% had SZ spectrum personality disorders, 34% had anxiety disorders and 32% had disruptive disorders. Duffy et al. (19) in 55 HRBP (average age of 17) observed around 50% of lifetime DSM-IV disorders. Wals et al. (20) also obtained an array of DSM-IV current diagnoses, several comorbid, in 44% of their HRBP adolescents, whereas Hillegers et al. (22) observed a rate of 59% of various disorders including mood disorders in adolescent subjects aged 16–26 years. Henin et al. (21) recently found a high rate of comorbid and non-comorbid disruptive and anxiety DSM-IV disorders in addition to mood disorders in HRBP adolescents. In all of the former studies that used a control group, the rates of diagnoses in controls were 20–30%.

### Non-diagnostic studies of behavioral and neurodevelopmental anomalies in young HRSZ

Most HR studies have reported non-diagnosed problem behaviors such as social withdrawal, aggressiveness, impaired relationships, attention problems and neuromotor deficits (14, 24). Poor social and school adjustment on one hand and developmental anomalies on the other hand are among the most commonly observed abnormalities in HRSZ. These two categories of abnormalities might be interpreted as vulnerability indicators (5, 6). Disruptive behavior in school was a predictor of later SZ appearance in the Copenhagen, Israeli and Helsinki HR studies (5, 25–27). Reviewing the HRSZ studies, Niemi et al. (5) and Owens and Johnstone (6) also observed the presence of neurocognitive deficits in terms of a poorer performance in several tasks encompassing attention (17, 28, 29), executive functions, memory and various aspects of neuromotor development (30–33).

### Non-diagnostic studies of behavioral and neurodevelopmental anomalies in young HRBP

Studies of HRBP are fewer than those of HRSZ (15, 16, 18). In childhood and adolescence, HRBP also showed behavioral and attentional problems (34–36) that did not look as specific to HRBP. In other words, HRBP would share many of their characteristics with HRSZ. In a meta-analysis, Lapalme et al. (37) found that over 50% of HRBP children presented behavioral problems compared with 29% of children of healthy parents, which is congruent with what was mentioned earlier. HRBP displayed elevated scores on every scale of the Child Behavior Checklist (CBCL) (38).

In summary, the diagnostic data accumulated so far in young offspring before the age of incidence of major psychosis would suggest similarities between HRSZ and HRBP in terms of high rates of non-psychotic disorders and high comorbidity early in life. Secondly, the same pattern of similarities seemed apparent in studies of behavior disturbances from rating scales. Thirdly, most studies had affected parents whose family history was not documented in details. Fourthly, we are aware of no studies having attempted a direct comparison of rates of clinical diagnoses between HRSZ and HRBP. Finally, in terms of the methodology, several limitations observed in previous studies of HRSZ and HRBP have led to the following recommendations (15): i) the use of the lifetime best estimate diagnostic procedure to define major psychosis in the parent; ii) documentation of presence or absence of a family history for the index parent; iii) the assessment of psychopathology in the spouse of the affected parent; iv) blindness of measurement to parental diagnosis. In the present study, we also added: i) a lifetime best estimate diagnosis procedure in the offspring including a direct structured interview; ii) a selection of affected parents who descended from thoroughly assessed and densely affected kindred to assure that we dealt with the genetic form of the illness; and iii) the use of the same methods and measurements for the concurrent study of the HRSZ and the HRBP.

### Aims of the study

We examined, before the age of incidence of major psychosis, the rate of any DSM-IV diagnoses in young offspring with an extreme genetic loading. Based on the increasing evidence that numerous phenotypic characteristics in adulthood are shared by SZ and BP, we also examined to what extent HRSZ and HRBP tended to have similar rates of diagnoses early in life.

## Material and methods

### Enrolled multigenerational pedigrees

The ascertainment of the sample of multigenerational families has been detailed in former reports (11, 13) and was performed in two waves of enrolment. We targeted all the multigenerational families densely affected by SZ or BP in the eastern Québec (Canada) catchment area. The first wave of kindred enrolment comprised 21 multigenerational families: six had 30–50 members, five had 20–29, seven had 10–20 and three had less than 10 family members. The average number per kindred was 26 members. The families had an average number of six members affected by SZ or BP. The sample consisted of seven SZ pedigrees (at least 85% of ill members affected by SZ or an SZ spectrum disorder, the remaining 15% having a BP spectrum disorder), six BP pedigrees (at least 85% of ill members affected by BP or a BP spectrum disorder, the remaining 15% having an SZ spectrum disorder) and eight mixed pedigrees, i.e. affected almost equally by both major psychoses. The high rate of mixed pedigrees may have resulted from our using a blind best estimate diagnosis. We have indeed demonstrated that unblind diagnosis had greater continuity with the most predominant diagnosis in a kindred than did blind diagnosis (12). Another possibility is that the size of our families may explain the mixture of some pedigrees: the larger the family, the more likely it can become mixed. The mean age of onset of adult family members was 25.4 (SD 8.5) years for SZ and 28.8 (SD 10.3) years for BP. The mean age at evaluation was 43.8 and 56.4 years respectively (13).

### Sample of offspring

The ascertainment of kindreds was performed in two waves and the present offspring sample was drawn from the first wave of assessment. The first wave included 21 of the 48 kindreds and led to the identification of 54 offspring aged between 7 and 22 years (mean 17.5) who belonged to the most proximal generations. These offspring had one parent affected by DSM-IV SZ or BP who was a member of a kindred. The description of the sample is in Table 1. The offspring were administered a lifetime best estimate diagnosis as described later. Ninety-three per cent of the contacted parents and subjects agreed to participate. The study was explained and a signed consent was obtained, as reviewed by our University Ethics Committee. The two parents of the HRSZ and HRBP as well as their unaffected spouses had been administered a consensus lifetime best estimate DSM diagnosis quite similar to that applied to all previous adult pedigree members (11, 12).

**Table 1:** Offspring sample characteristics

	Total sample of HR	HRSZ	HRBP
Number of HR	54	28	26
Mean age of HR* (years)	18.6 ± 4.5	18.4 ± 4.4	18.7 ± 4.6
Gender of HR, *n* (%) males	25 (46.3%)	10 (35.7%)	15 (57.7%)
Mean CGAS of primary Dx of HR	65.67 ± 16.06	62.68 ± 17.69	68.79 ± 13.86
Mean GAS of parents†	67.63 ± 13	60.35 ± 12.86	75.72 ± 7.18

* Mean age at evaluation.

† Parental average GAS measured during the intervals between acute episodes across life.

### Clinical assessments

DSM-IV diagnoses in the offspring were made blind to the parent diagnosis by means of a lifetime best estimate procedure reviewing all available medical records, family informants, interviews and the structured interview with the offspring or the parent. Blindness to parent’s diagnosis was assured by editing all the information related to the parent’s diagnosis. Then MM, MAR, NG and a senior research psychiatric nurse, who were blind to parents’ diagnosis (as they were not involved in the data gathering) reviewed the available information and made a consensus DSM-IV diagnosis of primary diagnosis and comorbid diagnoses. The K-SADS (39) was administered with the parents and the children for subjects under 18 years of age, and the SCID (40) with the subjects over 18 years of age. For assessing the global severity of diagnosis in offspring, the Children Global Assessment Scale (CGAS) (41) was used in subjects under 18 years of age and the Global Assessment Scale (GAS) (42) in those more than 18 years of age. In the affected parents, the GAS was also used to measure the severity and the social functioning during the intervals between acute episode across the entire life according to a method we reported elsewhere (12).

### Statistical analysis

When appropriate, the distributions of DSM diagnoses were compared by means of chi-squared statistics. The comparisons of CGAS or parental GAS scores in HRSZ and HRBP were made by means of *t*-tests.

## Results

Tables 2–4 provide the detailed description of the DSM-IV axes I and II diagnoses. In subjects presenting more than one diagnosis, the primary diagnosis was the one judged to be the most contributory to impairment. In around 60% of the HRs presented, at least one non-psychotic clinical diagnosis warranting a consultation and a high rate of comorbid disorders was observed. Despite the relatively small sample size, some observations can be cautiously derived from the data. The inspection of Tables 2–4 did not reveal differences between HRSZ and HRBP in terms of the proportion of subjects having more than one diagnosis (respectively 12 of 17 and 11 of 19 had more that one diagnosis, *χ*^2^ = 0.63, d.f. = 1, *P* = 0.43). When the subjects with DSM learning and communication disorders were excluded, the proportion of subjects having a diagnosis was similar in HRSZ and HRBP (*n* = 12 and 16, respectively, *χ*^2^ = 0.23, d.f. = 1, *P* = 0.64). DSM learning and communication diagnoses showed a potential higher trend in HRSZ (6/19) than in HRBP (1/17; *χ*^2^ = 3.78, d.f. = 1, *P* = 0.05). The ADHD diagnoses (*n* = 8) as primary or secondary diagnoses appeared rather spread among the two groups. The CGAS scores of the HRSZ (mean 62.9; SD 17.7) were, on average, six points lower than those of HRBP (mean 68.8; SD 13.9) but the difference was not statistically significant (*t* = 1.34, d.f. = 47, *P* = 0.19). The average age of onset was 10.0 years (SD 4.8) in the whole HR sample: 9.9 years (SD 4.1) for HRSZ and 10.2 years (SD 5.6) for HRBP.

**Table 2:** Description of lifetime DSM-IV diagnoses and comorbidity in offspring of SZ (*n* = 28) parents according to axis I and axis II

		Primary diagnosis		Secondary diagnosis
			
	Subject number	Axis I behavior disorders (*n* = 18)	Severity*	(Axes I and II disorders)
Mood disorders	4715 (F)	Major depressive disorder recurrentunspecified (296.30)	51	Avoidant personality disorder (301.82) (axis II)
	4726 (F)	Bipolar disorder NOS (296.80)	50	Attention-deficit/hyperactivity disorder NOS (314.9)
				Other (or unknown) substance abuse (305.90)
				Borderline personality disorder (301.83) (axis II)
	4740 (F)	Major depressive disorder,single episode,unspecified (296.20)	40	Social phobia (300.23)
				Other (or unknown) substance abuse (305.90)
				Enuresis (not due to a general medicalcondition) (307.6)
Anxiety disorders	4701 (M)	Anxiety disorder NOS (300.00)	55	Specific phobia (300.29)
				Enuresis (not due to a general medicalcondition) (307.6)
	4721 (M)	Panic disorder withagoraphobia (300.21)	40	Anxiety disorder NOS (300.00)
				Agoraphobia without history of panicdisorder (300.22)
				Other (or unknown) substance abuse (305.90)
	4738 (F)	Specific phobia (300.29)	50	Anorexia nervosa (307.1)
				Bulimia nervosa (307.51)
	4739 (M)	Separation anxiety disorder (309.21)	55	–
	4745 (M)	Separation anxiety disorder (309.21)	50	–
Substance-related disorders	4729 (F)	Other (or unknown) substanceabuse (305.9)	60	Separation anxiety disorder (309.21)
				Specific phobia (300.29)
Attention-deficit anddisruptive behaviordisorders	2969 (M)	Attention-deficit/hyperactivitydisorder, combined type (314.01)	40	Oppositional defiant disorder (313.81)
				Enuresis (not due to a general medicalcondition) (307.6)
				Disruptive behavior disorder NOS (312.9)
				Learning disorder NOS (315.9) (axis II)
	4725 (F)	Attention-deficit/hyperactivitydisorder NOS (314.9)	45	Oppositional defiant disorder (313.81)
				Other (or unknown) substance abuse (305.90)
				Separation anxiety disorder (309.21)
				Schizotypal personality disorder (301.22) (axis II)
	2962 (F)	Oppositional defiant disorder (313.81)	N/A	Specific phobia (300.29)
Learning and communicationdisorders	2967 (F)	Learning disorder NOS (315.9)	N/A	–
	2968 (F)	Learning disorder NOS (315.9)	51	–
	4712 (M)	Learning disorder NOS (315.9)	N/A	–
	4784 (M)	Expressive language disorder (315.31)	75	–
	4785 (M)	Expressive language disorder (315.31)	75	–
Others	4728 (F)	Bulimia nervosa (307.51)	50	Other (or unknown) substance abuse (305.90)

	Subject number	Axis II (*n* = 1)		Axes I and II disorders
	
Personality disorders	4769 (F)	Personality disorder NOS (301.9)	40	–

Subjects without a diagnosis (*n* = 9): 2970 (F), 4703 (M), 4705 (F), 4713 (F), 4716 (F), 4730 (F), 4732 (F), 4775 (M), 4821 (F). F, female M, male.

* The severity at the time of occurrence of the primary diagnosis as assessed by the CGAS for subjects under 18 years of age and by the GAS for subjects more than 18 years of age.

**Table 3:** Description of lifetime DSM-IV diagnoses and comorbidity in offspring of BP (*n* = 26) parents according to axis I and axis II

		Primary diagnosis		Secondary diagnosis
			
	Subject number	Axis I behavior disorders (*n* = 17)	Severity*	(Axes I and II disorders)
Mood disorders	4734 (F)	Bipolar disorder NOS (296.80)	45	Alcohol abuse (305.00)
				Other (or unknown) substance abuse (305.90)
	4796 (M)	Bipolar disorder NOS (296.80)	40	Other (or unknown) substance dependence (304.90)
Anxiety disorders	4710 (F)	Anxiety disorder NOS (300.00)		Specific phobia (300.29)
	4723 (F)	Separation anxiety disorder (309.21)	70	–
	4744 (F)	Panic disorder without agoraphobia (300.01)	60	–
	4750 (M)	Separation anxiety disorder (309.21)	N/A	Encopresis, without constipation and overflowincontinence (307.7)
				Tic disorder NOS (307.20)
Substance-relateddisorders	4754 (F)	Other (or unknown) substance abuse (305.90)	71	–
	4758 (F)	Poly-substance dependence (304.80)	60	Alcohol abuse (305.00)
				Disruptive behavior disorder NOS (312.9)
	4771 (M)	Alcohol dependence (303.90)	60	Other (or unknown) substance abuse (305.90)
	4772 (M)	Alcohol dependence (303.90)	50	Other (or unknown) substance abuse (305.90)
Attention-deficit anddisruptive behaviordisorders	4748 (M)	Attention-deficit/hyperactivitydisorder NOS (314.9)	70	Tic disorder NOS (307.20)
	4751 (M)	Attention-deficit/hyperactivitydisorder NOS (314.9)	N/A	Separation anxiety disorder (309.21)
				Encopresis, without constipation and overflowincontinence (307.7)
				Tic disorder NOS (307.20)
				Obsessive-compulsive disorder (300.3)
	4755 (M)	Attention-deficit/hyperactivity disorder,combined type (314.01)	60	Oppositional defiant disorder (313.81)
	4777 (M)	Attention-deficit/hyperactivitydisorder NOS (314.9)	55	Enuresis (not due to a general medical condition) (307.6)
Learning disorders	4724 (M)	Learning disorder NOS (315.9)	50	Attention-deficit/hyperactivity disorder NOS (314.9)
Others	4736 (M)	Tourette’s disorder (307.23)	70	–
	4779 (F)	Transient tic disorder (307.21)	70	–

Subjects without a diagnosis (*n* = 9): 4707 (M), 4709 (M), 4746 (M), 4759 (F), 4762 (F), 4767 (F), 4781 (M), 4782 (F), 4820 (M). F, female M, male.

* The severity at the time of occurrence of the primary diagnosis as assessed by the CGAS for subjects under 18 years of age and by the GAS for subjects more than 18 years of age.

**Table 4:** Proportions of lifetime DSM-IV diagnoses in the whole high-risk (HR) sample and in offspring of schizophrenia (HRSZ) and bipolar (HRBP) parents

	Total HR sample (*n* = 54)	HRSZ (*n* = 28)	HRBP (*n* = 26)
Schizophrenia	0	0	0
Schizophrenia spectrum disorders*	1 (1.9)	1 (3.6)	0
Schizotypal personality disorder	1 (1.9)	1 (3.6)	0
Any affective disorders*	5 (9.3)	3 (10.7)	2 (7.7)
Major depressive disorder	2 (3.7)	2 (7.1)	0
Bipolar disorder	3 (5.6)	1 (3.6)	2 (7.7)
Any anxiety disorders*	20 (37.0)	13 (46.4)	7 (26.9)
Anxiety disorder NOS	3 (5.6)	2 (7.1)	1 (3.8)
Specific phobia	5 (9.3)	4 (14.3)	1 (3.8)
Social phobia	1 (1.9)	1 (3.6)	0
Agoraphobia	1 (1.9)	1 (3.6)	0
Separation anxiety disorder	7 (13.0)	4 (14.3)	3 (11.5)
Obsessive-compulsive disorder	1 (1.9)	0	1 (3.8)
Panic disorder (with or without agoraphobia)	2 (3.7)	1 (3.6)	1 (3.8)
Any disruptive disorders*	14 (25.9)	7 (25.0)	7 (26.9)
Attention deficit disorder	8 (14.8)	3 (10.7)	5 (19.2)
Oppositional defiant disorder	4 (7.4)	3 (10.7)	1 (3.8)
Disruptive behavior disorder	2 (3.7)	1 (3.6)	1 (3.8)
Any personality disorders*	3 (5.6)	3 (14.3)	0
Personality disorder NOS	1 (1.9)	1 (3.6)	0
Avoidant personality disorder	1 (1.9)	1 (3.6)	0
Borderline personality disorder	1 (1.9)	1 (3.6)	0
Learning disorders*	5 (9.3)	4 (10.7)	1 (3.8)
Learning disorder NOS	5 (9.3)	4 (14.3)	1 (3.8)
Communication disorders*	2 (3.7)	2 (7.1)	0
Expressive language disorder	2 (3.7)	2 (7.1)	0
Other disorders*	30 (55.6)	12 (42.9)	18 (69.2)
Alcohol and substance abuse and dependence	16 (29.6)	6 (21.4)	10 (38.5)
Eating disorder (bulimia/anorexia)	3 (5.6)	3 (10.7)	0
Tic disorder	4 (7.4)	0	4 (15.4)
Tourette’s disorder	1 (1.9)	0	1 (3.8)
Enuresis and/or encopresis	6 (11.1)	3 (10.7)	3 (11.5)

Values in parentheses are percentage.

* Because several individuals had more that one lifetime disorder, the number of individual diagnoses does not necessarily add up to the total number of subjects for a category.

As the diagnoses of the parent, SZ or BP, did not appear strongly associated with the clinical status of the offspring, we also looked at the severity of parental illness. The severity of impairment in the parent, as indexed by the GAS score (11, 12), was similar in the HR group with a diagnosis as in the one without (mean parental GAS, respectively, of 64.8 and 67.8; *t* = 0.65, *P* = 0.52).

As some of the SZ and BP parents were from mixed kindreds and thus had a family history of both major psychoses, we regrouped the HRs into those having a SZ parent from a SZ kindred (*n* = 9 offspring), those having a BP parent from a BP kindred (*n* = 20 offspring) and those with a parent from a mixed kindred (*n* = 25 offspring). We recompared this new category of HRSZ (*n* = 9) to the new category of HRBP (*n* = 20) and found that the rates of comorbid diagnoses remained the same in each group (*χ*^2^ = 0.50, d.f. = 1, *P* = 0.63) and we observed again an absence of difference in terms of the absence or presence of disorders (*χ*^2^ = 1.77, d.f. = 1, *P* = 0.37). In this new analysis, however, the CGAS score in HRSZ from a SZ kindred tended toward a greater severity (mean 53.0; SD 13.5) compared with that of the HRBP from a BP kindred (mean 66.1; SD 15.2, *Z* = −1.87, *P* = 0.06).

## Discussion

### Limitations of the study

Several limitations possibly affecting the results and their interpretation need to be discussed. First, the small sample sizes may have favored type II errors and may have prevented us from seeing more quantitative differences between the HRSZ and the HRBP in more specific diagnostic categories. The second limitation is the extent to which this very high-risk sample is representative of general samples of SZ or BP patients and their children, requiring precaution before generalizing the present results to the offspring of an ill parent in the general population. However, despite the high likelihood that the affected parents present the familial form of illness, the possibility of some sporadic forms of disease cannot be eliminated. Thirdly, our goal was to look at similarities and differences among the offspring at extreme risk of SZ or BP. Even though we report a rate of diagnosed disorders that is very similar to those recently reported in different populations of offspring at risk, we had no normal controls for the comparison of the DSM diagnoses. Fourthly, one has to remember that the present offspring had not reached the age of incidence of major psychosis and that this must be considered when making comparisons with other studies of HRs in adulthood. Nonetheless, several observations can be derived from the data.

### Rates of clinical diagnosis and type of disorders

The present 60% rate of non-psychotic clinical disorders in these young HR offspring having a very dense family history is very consistent with the high rates of diagnosed disorders reported in several recent reports of HRSZ or HRBP in childhood, adolescence and postadolescence (14–16, 18–23). In most of these studies, however, the presence of a major psychosis in the first- or second-degree relatives of the parent was not documented and, consequently, the index parent might have been either familial or sporadic (non-familial form of the disease).

Even though our HRs have not yet reached the age of incidence of psychosis, it is informative to consider the studies having followed up HRs until adulthood such as the Swedish HR study (9, 43). In 22-year old HRs, they found a lifetime rate of any axis I DSM-III-R of 54% in HRSZ and 41% in HRs of mothers having affective psychosis, and a distribution of specific diagnosis quite similar to ours in addition to a sizeable level of comorbid diagnoses. The Helsinki study of HRSZ provided a 40-year follow-up and observed a 23% rate of any axis I disorders but the authors acknowledged that this rate might have been higher had they interviewed the offspring, the diagnosis having been based on medical record reviews only (8). After 15–42 years of follow-up, 20% of the HRSZ of the Copenhagen HR study had developed a SZ spectrum disorder and an additional 20% presented a lifetime non-psychotic DSM-III-R disorder with high level of comorbid disorders (10). The British birth cohort follow-up is also of interest with regard to the present findings. Jones et al. (44) observed that the subjects who later developed SZ presented more internalized and externalized behavior disorders, especially anxiety-like behavior, assessed by means of rating scales. In the same cohort, the subjects who later developed adult affective disorders as indexed by the Present State Examination (PSE) displayed, in childhood, withdrawing behavior as rated by teachers (45). The subjects of the Dunedin birth cohort who, at the average age of 26 years, developed a schizophreniform disorder were more likely to present internalizing behavior problems before the age of 11 years as rated by parents and teachers (46). Hence, risk studies and birth cohort follow-ups, along with the present findings, indicate that behavior disorders or comorbid non-psychotic disorders appear to be precursors of major psychosis in adulthood, which may have implications for prevention, especially in the children and adolescents having a family history of major psychosis.

Even though the present HRs had their origin in densely affected kindred that were well documented epidemiologically (2, 12, 13), our HRs did not tend to present a higher rate or more severe clinical syndromes than the HRs in the former studies (14, 21, 22). Very few studies have given attention to the relationship between the familial loading in first- and second-degree relatives of the affected parent and the severity of clinical status in the offspring. Very recently, Reichart et al. (7) observed in HRBP, aged 12–21 years, that a high number of relatives of the BP parent who were affected by unipolar depression was associated with a higher severity of offspring internalized and externalized symptoms on the CBCL scale. As regards our study, it suggests that the offspring of a SZ or BP parent descending from such dense families would not display a higher rate of disorders or more specific risk of non-psychotic disorders early in life than the offspring of an ill parent with a lesser or no familial loading, although we did not assess offspring of a parent with sporadic illness to provide a direct comparison. The limited power due to the small sample size may have prevented us to observe the presence of specific but less frequent disorders.

### Clinical similarities in HRSZ and in HRBP

The nature of the observed disorders is also worth a few comments. Five HRs, around 9.3%, had already developed a mood disorder. As observed in former HRSZ and HRBP studies assessing diagnoses, we found both internalized-anxiety and externalized-disruptive disorders in the whole HR sample in proportions that were quite similar in HRSZ and HRBP, and a high frequency of comorbid disorders (14, 16, 18, 23). The ADHD diagnoses also were rather spread among our two risk subgroups. Overall, HRSZ and HRBP tended to display rather similar characteristics in terms of the rate of non-psychotic diagnoses, the type of diagnoses, their severity level, the high comorbidity and the age at onset of diagnosed disorders. The only potential difference could be that the HRSZ with a parent having a family history of SZ only, vs. the SZ parents having a family history of both SZ and BP, might have a higher overall rate of disorder. This calls for further investigation as most HR studies did not distinguish whether the affected parent was sporadic or familial, and consequently cannot be informative about the effect of the family history of the parent on the clinical status of the offspring.

Also, when we look at the clinical status of our HR offspring, especially in light of the other HR studies and birth cohort follow-ups, it is obvious that HR children and adolescents present non-psychotic disorders warranting a consultation, that the diagnoses are various and often comorbid, and that such early disorders increase the risk of major psychosis in adulthood. The onset of these non-psychotic disorders often occurs before adolescence, both males and females are affected, and anxiety-like disorders may be salient, according to several studies, as are diagnoses of externalized behavior. What is definitive is that social functioning is affected in these children at risk and that more preventive research and specialized group intervention targeting these HR offspring should deserve attention as these youngsters represent 2–3% of our children.

An increasing number of reports on adult SZ and BP patients and their adult relatives show that several phenotypic characteristics appear common to the two disorders (2, 4, 13, 47), along with a large number of shared susceptibility loci now replicated in numerous linkage studies (4, 47) including ours in these kindreds (13). There is a debate as to whether HRSZ and HRBP have specific developmental precursors or if there are only quantitatively greater effects in HRSZ (1, 3). In this line of continuity, our findings would suggest that, at an early age, when the risk mechanisms are in play, the HRSZ and the HRBP already have some common phenotypic features, except perhaps that HRSZ might present a slightly more severe clinical picture especially if the offspring has a rather homogeneous family history of SZ instead of a mixed history of SZ and BP. The commonalities between the two major psychoses might take root in the early pathogenesis, as others have also hypothesized (1), assumedly implicating genetics and/or environmental factors that need to be further investigated in high-risk research. This potential lack of specificity is challenging not only in terms of nosology but also in terms of public health implications.
